# Intersubjectivity as an analytical concept to study human-animal interaction in historical context: street dogs in Late Ottoman period

**DOI:** 10.3389/fsoc.2024.1389010

**Published:** 2024-06-19

**Authors:** Burak Taşdizen, Erman Örsan Yetiş, Yekta Bakırlıoğlu

**Affiliations:** ^1^Design, Technology, and Society, Graduate School of Social Sciences, Özyeğin University, Istanbul, Türkiye; ^2^Department of Politics and International Relations, University of Sheffield, Sheffield, United Kingdom; ^3^ImaginationLancaster, Lancaster University, Lancaster, United Kingdom

**Keywords:** intersubjectivity, human-animal interaction, animal history, co-existence, joint attendance, anthropomorphism, generative iterability, indistinctive boundaries

## Abstract

Human knowledge pertaining to human-animal interaction is constructed by the human author, albeit the presence of animal subjects. Such a human lens is pronounced when studying human-animal interactions across history, whose nonhuman animal subjects are not only absent, and therefore eliminating the possibility of conducting empirical studies *in situ*, but also their experiences are filtered by the interpretative lens of human authors of extant historical accounts as well as contemporary human analysts who interpret these accounts. This article draws upon such epistemological limitations of understanding nonhuman animal presence in historical accounts and offers human-animal intersubjectivity as an analytical concept, involving generative iterability and indistinctive boundaries that emphasise intersubjective openness and relationality, to trace and disclose the continuity of human-animal co-existence. The article’s historical scope is the Late Ottoman period characterised by a sense of temporal and spatial disorientation and reorientation for humans as well as street dogs during its modernisation processes.

## 1 Introduction

They have the most lunatic hopes, these beasts; they are just fools, utter fools. That’s why we like them; they are our dogs; finer dogs than any of yours (Kafka, 1971, 447).

In an abandoned warehouse, two lovers, a concubine, Safiye, and a black eunuch, Nadir, both of whom previously served their imperial Sultan Abdulhamid II at his harem and were later freed, are sleeping by the fire they made to the distant sounds of crying and howling dogs. Earlier, the Sultan was deposed by the Committee of Union and Progress, the harem was dissolved, and the concubines along with other servants were freed. The concubines who had relatives or distant acquaintances sought refuge in their kin, while those who were brought from distant lands had no one and nowhere to turn to. In the morning, the concubine encounters a female street dog, as orphaned as the lovers. The dog, Gülfidan the narrator reminds the audience, must be one of the few remaining dogs to have managed to escape the deportation to a deserted, barren island near the city, from where the sounds of dogs’ painful cries were carried by the wind.

The above-mentioned scene depicts one of the final scenes of *Harem Suare*, directed by Ferzan Özpetek (1999). The fictive, historical juxtaposition^1^ of the overthrowing of the Ottoman Sultan Abdulhamid II in April 1909 and the exile of street dogs in July 1910 to the island of Oxias [*Hayırsızada*], as well as the brief interaction between the concubine, the eunuch and the dog illustrate how, once at the service of the now-overthrown Sultan, both the concubine and the eunuch have now become strays just like this dog. All these depicted characters in the scene could be said to occupy a liminal position in society, for they “do not belong” any longer, therefore, seeking temporary refuge in an abandoned setting. The concubine, the eunuch and the dog tell each other the end of a story. The scene is thought-provoking in terms of how such an interspecies encounter could narrate evoked feelings of disorientation and fear amid uncertainty. The contiguous life of the human and the dog holds the possibility of unexpected encounters, untold stories and emotions emerging and turning into stronger expressions. The turn of the 20^th^ century in the Ottoman Empire, as the scene depicts, could be argued to harbour ambiguity and a sense of disorientation and reorientation for those who experienced it, as if in the middle stage of a rite of passage, for the dissolution of the Empire marked the ending of one era, and the beginning of another, leading to how certain lives were made disposable, without acknowledging the very mechanisms (slavery and domestication, respectively^2^) that fostered their dependency upon Man and shouldering the responsibilities thereof.

Experiences of interspecies liminality exemplify ways in which humans and nonhuman animals^3^ may form intimate relationships across species divide (Taşdizen, 2024). Shared experiences of liminality intertwined with interdependency and uncertainty depicted above pave the way toward a novel and comprehensive understanding of human-animal interaction based on a conceptualisation of intersubjective relationality that is open, dynamic, and indistinctive. Accordingly, this article contemplates human-animal coexistence by joining recent post humanist approaches which critique the subject-object dichotomy, one strand of which manifests itself in the historical narratives pertaining to animals as “surrogates for theory” (Haraway, 2003, 5), only to be utilised in support of making sense of grand human narratives such as origins, culture, religion, or modernisation. Such narratives that are based on a human-animal dichotomy provide a rather limited notion of intersubjective relationality.

Human-animal coexistence not only creates emotional/affective repercussions as in emotional contagion or social cognition, as emphasised in various accounts on the nature of human-animal interaction (e.g., Leconstant and Spitz, 2022), but also lays the foundations for humans to make sense of their human selves through animals (Haraway, 1991). However, this should not be regarded as a mere one-way interpretation taking animals as symbolic resources (e.g., Turner, 2013), through which only the human subject may be understood as the symbolic actor. It could also become a way of understanding the animal other. In this article, we explore the potential of deploying intersubjectivity as an analytical concept to examine human-animal interaction in the historical context of the Late Ottoman period to provide an alternative lens that brings forward the subjectivity of animals through their intersubjective relationality with humans.

## 2 Theoretical background

### 2.1 From innate anthropocentrism to de-centring of the human: Approaches to study the animal

Drawing on Calarco’s robust and nuanced arguments, the studies on animals elongated on a spectrum from across animal welfare to animal liberation could be clustered under three main approaches: (1) identity, (2) difference, and (3) indistinction (Calarco, 2011, 2015). The *identity* approach is characterised by its emphasis on shared traits across species through the critique of binary distinctions sharply drawn between humans and animals (Calarco, 2011, 42). However, while the identity approach criticises the blatant forms of logocentrism, it still attributes a unique position to human logos in extending or withdrawing moral identity to animals. In doing so, an implicit form of the same anthropocentric logic prevails and is extended to animals. Deriving from contemporary normative theory, this approach is rather limited by its atomistic-individualistic notion of ethics centred around personal, moral responsibility and individual consumer-based market solutions. Thus, identity approach remains rather limited in what it could offer for transformative or political action in pursuit of social justice for animals. Drawing upon critical animal studies (e.g., Cassidy, 2002; Alger and Alger, 2003; Hurn, 2012), this would require intersubjective relationality to appreciate animals’ subjectivity, which can enable mutual recognition and joint attendance^4^ instead of featuring animals only as an extension of human logos, and thus, subject of human moral concern.

The *difference* approach (henceforth, différance) mostly characterised by Derrida’s (2016) work, seeks a way out of anthropocentrism that is at play in the identity approach. For Derrida, the human logos, which inevitably establishes a domination framework in favour of the human, could never fully comprehend the worlds of animals (Ertuğrul, 2015). By arguing against a neatly described and hierarchically defined human/animal distinction, Derrida complicates both categories within themselves. In that sense, Derrida not only provides underlying arguments for critical approaches that challenge the notion of species as an isolated and frozen category (Haraway, 2003; O'Connor, 2013, among others) but also provides a novel way to understand human-animal relationality other than the notion of granting animal moral status based on its proximity to human logos. Having said that, Calarco (2011) rightly argues about the limitations of the difference-based approach regarding its overemphasis on the radical difference between human and animal categories as well as among members of the same species. According to Calarco, Derrida’s *différance*, too, falls short in terms of any political prospect it could offer for a social justice struggle for animals (Calarco, 2011). Here, it should be noted that Derrida’s real intention was not to directly engage in animal liberation, but rather to provide a deconstructivist critique against logocentrism which is the main pillar of Western conceptualisation of humanity (Ertuğrul, 2015).

Derrida’s arguments on *différance*, however, may potentially lead away from anthropocentric tendencies and pose new directions. *Différance* refers to how meaning is never stable but ever-shifting depending on context-specific interpretations mediated through various means such as thought, language and writing. Thus, *différance* involves an interplay through *generative iterability* in different contexts (Butler, 2011). This may help recontextualise the rights belonging to the human beyond the human and toward the animal, rather than simply extending them as in the identity approach. We use Derrida’s *différance* to strategically pervert anthropomorphism from anthropocentrism, and to excavate remnants of what is denied, forgotten, and excluded from such anthropocentric narratives pertaining to animals, which constitutes an undercurrent narrative of human-animal intersubjectivity. More promising for and relevant to intersubjective relationality, we argue, is Derrida’s concept of *arrivant* (Derrida, 1992), which could offer various possibilities and potentials to comprehend human-animal interaction. *Arrivant* refers to emergent phenomena which are never fully present but hold the potential to interrupt one’s mode of existence by bringing about transformative effects and be ethically forceful in different contexts. This concept challenges the notion of ethics solely limited to humans or sentient beings who are in social and/or biological proximity to humans (Calarco, 2011). We argue that *différance* and *arrivant* emphasise the interruptive nature of intersubjective relationality that harbours the potential for novel ways of re-interpretation, re-positioning, and re-articulation between different subjects, and thus, re-shaping of the subjectivities through this relationality beyond anticipated and preconceived ways.

The *indistinction* approach aims at suppressing any nostalgic desire to extend human traits to animals or any attempts to further complicate the differences between humans and animals (Calarco, 2011, 54). This is far from ‘elevating’ animals to the level of humans based on shared sentience and/or cognitive capacities (Calarco, 2011, 56). Rather, similar to ecofeminism, critical animal studies, and queer studies scholarship [Plumwood (2020) among others; i.e., Haraway (2003, 2008), and Braidotti (2013)], this approach attempts to recentre animal actors in such a way that the human no longer becomes the main reference point (Calarco, 2015). By doing so, the indistinction approach renders the human/animal distinction less absolute, by retrieving human’s privileged status. This approach, by acknowledging that the vast nonhuman world is marginalised by anthropocentric concepts and practices, brings forward zones of indistinction that harbour potentialities for alternative modes of relationalities. Such abandonment of anthropocentric distinction enables us to focus on enriching our understanding of human-animal interactions and to question whether animal justice may be grounded in a more nuanced understanding of such interactions. But, more importantly, indistinction approach also emphasises the coexistence and continuity of intersubjective relationality between humans and animals across space–time, and thus, history.

In this article, while we find both *différance* and *indistinction* approaches valuable respectively, we suggest a nuanced conceptualisation deriving from both in a creative manner that will allow us to better comprehend and appreciate the intersubjective relationality between the human and the animal. Hence, we propose the concept of *indistinctive boundaries* that can reflect both interdependence of and separation between the subjects, and that can create a space for the constitution of intersubjective relationality. Boundaries here stand for the difference and distance between subjects that constitute “inter,” thus, rendering subjects differentiable yet still relatable and then recognisable to each other. These constitute a *mediating space* which also harbours a character of indistinction since the boundaries can always be blurred, transgressed, and re-interpreted continuously but cannot and should not be denied, erased, or surpassed. Indistinctive boundaries underline the nature of intersubjective openness that we employ in this article.

### 2.2 Human-animal intersubjectivity and co-existence

Intersubjectivity, as originally defined by Husserl (2012), refers to the essential aspect of human existence that shapes the subject and the concept of an objective world. However, there seems to be a tendency to confuse intersubjectivity with Meadian social interactionism (Mead, 2015) which regards linguistic interactions as the basis for the development of an individual’s sense of self. This is attributed to the mastery of human’s capability of self-objectification, i.e., to regard not only the other but also the self as an object and act toward themselves in an interpretative manner (Turner, 2013). Turner argues that because it remains unknown whether and to what extent animals act toward themselves as objects, sociologists studying human-animal interaction should employ an approach that treats animals as *symbolic resources*, and not *symbolic actors*.^5^ This is because animals, according to Turner, cannot participate in intersubjective exchanges and constitute resources only to be utilised so long as they help understand human society. This reflects a rather autonomous, individualist, and even masculine understanding of the self that the human can craft, give a shape and then gain a sense of control over. Accordingly, this conceptualisation juxtaposes a highly limited understanding of intersubjectivity with a capability to regard the self as an object of (human) others that can pave the way for a shared understanding and intentionality. This truncated definition of the self undermines the potentiality of intersubjective openness by overemphasising so-called shared understanding and intentionality, which can only be the very products of such openness yet not the source of it. Self is hyphenated by other aspects, such as self-image, self-reflexivity, self-consciousness, and self-awareness, all of which reflect a peculiar anthropocentrism when appealed in narrow, abstract, and normative ways to search for a universal concept of human.^6^ Such a universal notion of human sets certain standards that work to exclude others, human or otherwise, by constructing them as “non” or “less than” human. These excluded others include animals, as well as people with disabilities who may lack such faculties for developing or maintaining the qualities of self, or racialised and sexualised others whose abilities toward themselves and others do not match with these qualities of self. Such a definition of intersubjectivity anchored around an anthropocentric mastery of self-objectification results in an ideal form of self-awareness, which we find highly limited and problematic. Instead, we argue that subjectivity is co-developed and (re)shaped through ongoing intersubjective processes and relationality with *indistinctive boundaries* that, in the first place, hinder the development and maintenance of such mastery over the self as an object. Hence we find Husserl’s original, broader formulation more promising in comprehending intersubjective relationality with all its complexity since it emphasises the *possibility* of developing understanding through exchanging one’s place with the other, which he describes as “trading places” [*Platzwechsel*], which may or may not lead to a shared understanding between subjects (Husserl, 1989). This complexity involves the indistinctive boundaries prior to shared understanding and intentionality, which, thus, have the decisive role in the constitution of such intersubjective relationality with its unpredictability and uncontrollability.

There is a wealth of evidence that convincingly showcases the complex cognitive abilities and consciousness of many animals (Hurn, 2012), as well as their non-verbal, embodied communicative capacities (Peltola and Simonen, 2024). Animals *do* influence human relations as active subjects and such multispecies interaction has been regarded as intersubjective (e.g., Cassidy, 2002; Alger and Alger, 2003; Hurn, 2012). Social animals are born with the ability to relate to others as creatures with social minds that develop through a dynamic co-determination of self and other, from which we conclude that animals are intersubjectively open (Thompson, 2001, 3). Intersubjective openness indicates the generative role of *iterability* involving anthropomorphism but it is not limited to it. It may also pave the way for *arrivant* that reflects the unanticipated dimensions of interaction between the subjects which inevitably both resist anthropocentrism and potentially go beyond anthropomorphic identifications and projections. Furthermore, intersubjective openness indicates *indistinctive boundaries* of co-determination of human and animal which can also elicit novel interpretations and re-articulations through such relationality. Accordingly, we argue that feminist psychoanalyst Jessica Benjamin’s conceptualisation of intersubjectivity is especially useful in recognising and explicating the interaction between human and animal subjects. Benjamin’s notion of intersubjectivity pertains to the idea that subjects are constituted by their relations with others, and that these relations are mediated by language, culture, and history (Benjamin, 1988). Through a critique of object-relational psychoanalysis, Benjamin advocates for a model of relating that is based on mutual recognition and respect whereby subjects can assert their autonomy and acknowledge their interdependence in tandem. Benjamin develops the concept of intersubjectivity mainly through looking at the relationship between infant, mother, and father, and criticises the model of dyadic relating based on complementarity in roles, with only one member of a dyad being a subject in a true sense while the other has to be reduced to an object. Despite her focus on infant, mother and father, Benjamin’s conceptualisation presents the potential to be extended into human-animal interactions, in which interdependence becomes a defining constituent of the relationship and may benefit our understanding of mediation among human and animal subjects. Human-dog relationship, the focus of this article, is especially fruitful as a topic to study interspecies interaction, supported by studies focusing on how dogs communicate and cooperate with their human companions by entering ‘interactional reciprocity’ similar to mothers and infants (Simonen and Lohi, 2021). In Benjamin’s account, mediation stands for both the context (as a form of a container of this continuing interaction between human and animal) and the ever-changing nature of the indistinctive boundaries that enable various forms of such intersubjective relationality. Intersubjectivity involves a mediating space between two subjects, as well as the temporal and spatial context in which these subjects have historically been shaped through an ongoing interdependence, co-evolving. However, interdependency, both between subjects and between the subjects and the context that they depend on, is far from indicating a deterministic character. While interdependency shapes intersubjective relations, it should be regarded as a medium that harbours various potentialities. This lens paves the way to comprehend interactions and relationality not only in empirical observations but also in different historical periods and varied geographical contexts, through human narratives.

An example of such an intersubjective and interdependent relation across species is humans’ relations to domestic^7^ animals, and, in this article, dogs, who constitute our central focus. Haraway (2003) develops her concept of *companion species* to argue against neatly isolated categories of human and animal, and argue for historically entangled co-evolutions of humans and domestic animals, in which neither precedes the other. To Haraway, humans and dogs owe each other their current existence. She traces the human-animal relationship back to the earliest human-dog encounter and overturns the conventional narratives on evolutions of the human and the dog, as well as the domestication of the latter. Haraway (2003) argues that co-evolution occurs through the interaction of species, and avoids the narrative of domestication as Man’s dominance over the animal to docility. Human-dog relationship, as well as many other histories of domestication across various sites (Zeder, 2012b), varies according to the historical and social context and does not have a fixed character, but such human-animal interactions often include ongoing practices of care and tending to the needs of the animal (Losey, 2022, 138). A recent study on dog ownership in the UK confirms that, in spite of the care work that goes into maintaining human-dog relationship, both parties benefit from their relationship in terms of health and wellbeing as well as emotional attachment (Anderson et al., 2024). It is in such attentive and interdependent relationships that dogs benefit from their own domestication processes. Such a perspective on domestication allows us to see dog’s subjectivity through its active agency in shaping relations to humans. In a similar vein, anthropologist Tsing (2012, 144), too, emphasises the interdependency of companion species, and argues against the prevailing assumption of human as an isolated entity. To Tsing, human is an interspecies relation.

We argue that human-dog interaction should be understood in its historical complexity, where both parties have taken part in a co-evolution process, shaped by and disclosing the subjectivities of both. In this endeavour, we find Benjamin’s conceptualisation of intersubjectivity especially promising as an analytical concept, and we translate it into human-animal interaction by revoking Husserl’s original definition that suggests intersubjectivity beyond meaning-oriented interaction. Building upon Calarco’s *indistinction* and Derrida’s *différance*, *iterability* and *arrivant*, we developed our concepts of *generative iterability* and *indistinctive boundaries* to recognise and reveal intersubjective openness and relationality, encompassing the mutual dependency between and co-evolution of companion species that are humans and dogs. In this way, we can recognise the diverse agencies of both humans and animals, and the historical and material conditions of their shared lives. In this paper, we attempt to demonstrate how this might contribute to a more nuanced understanding of human-animal interaction as intersubjective and interdependent.

## 3 Methodology

Studying human-animal interaction across history cannot rely on first-hand observations of such interactions, in comparison to contemporary forms of human-animal interaction which may rely on sets of observations of animals *in situ*. Instead, historical approaches to studying the animal often draw upon extant human narratives pertaining to animals, which can sometimes be supported or disproved by anthropological or archaeological evidence. Cognisant of the impossibility of eliminating the human lens, when empirical observation of the human-animal interaction as a part of data collection is simply not possible, this article poses the following questions:

- How can we study human-animal interaction in historical accounts through the lens of intersubjectivity?
- How do we uncover the continuous and ever-changing nature of intersubjective relationality and co-existence of humans and animals in extant accounts produced and transmitted by human subjects?

To explore these questions, this article draws upon the historical narratives of human-dog interaction in Late Ottoman period, thereby providing a case from the periphery of the ‘West’ both in terms of geography and history. A review of historical accounts on the decanisation^8^ process of Istanbul shows that the extant literature focuses mostly on either providing interpretations of ‘authentic’ Ottoman culture or making sense of human phenomena such as modernisation, public health, and urban transformation, as we will illustrate in our discussion. We argue that dogs do not merely constitute symbolic resources to help justify or challenge recycled cultural and historical narratives concerning the city, but they are subjects who enter intersubjective relations with humans.

In this endeavour, we unavoidably encounter anthropocentric features involving anthropomorphic identifications, remembrance, and affect. However, it may not be possible to find a way to completely escape the human lens in understanding and representing the animal experience, and we, the human authors, cannot claim to write from the animals’ standpoints, which are vastly different from and alien to ours (Horsthemke, 2018, 210). It is inevitably through the lens of the human that we narrate and interpret the realities of animals, but we can still recognise the potential to identify intersubjective relationality and co-existence of humans and animals in historical contexts considering the generative power of the *iterability* of these historical and cultural narratives, rather than regarding these features as pitfalls that prevent to reveal this relationality.

In this article, we benefit from Jessica Benjamin’s (1988) expanded notion of *mediation*, in order to understand how different forms of communication and representation shaped human-dog interactions, and how these relations reflected or challenged the dominant ideologies and discourses of different periods. We utilise (Haraway, 2003) *companion species* to understand how human and dog lives were intertwined and interdependent, and how these interactions created or transformed the naturecultures of Ottoman and modern Turkey. Through this lens, we investigate the intersubjective relationality among and co-existence of humans and dogs by looking into shared urban spaces in the historical context of Istanbul where both care and violence practices of humans were performed. Our historical scope covers the Late Ottoman Era, during which modernisation efforts pertaining to spatial reorganisation were intensified. In this article, we inquire into historical secondary sources providing travelogues of Western travelers originally written in English, German and French, as well as texts taken from Ottoman popular media in Ottoman Turkish and English, and articles and diaries of Turkish-speaking intelligentsia of the period. We rely on the translations of these sources either into modern Turkish in Latin alphabet or into English. We selected these sources thematically (i.e., featuring human and dog relationships, or analyses of dogs’ lives in urban contexts) and within a historical scope (i.e., the Late Ottoman period). Because such resources on human-dog interaction are scarce, we used a collection of sources in a way that corresponds to each other and concretises our hypothesis. We co-collected and co-analyzed these historical accounts and narratives using textual analysis by which we sought to reveal connections between different texts and the context they were written and to derive novel interpretations and meaning from them in terms of intersubjective relationality we propose. All three authors of the article feature outsider qualities to dog experience but are scholars who are insiders in the Turkish context.

## 4 Interpretation of human-dog co-existence as an extension of Ottoman culture

Dogs in the Ottoman Empire are often mentioned in the travel accounts of Western visitors in the late nineteenth and early twentieth centuries. Some of these accounts describe dogs as scavengers, nuisances, or even dangers to the travelers, while others demonstrate more sympathy or admiration for the animals. However, their observations and expressions mostly take animals as symbolic resources to recount their orientalist interpretations that mainly attribute authenticity and even exoticism to Ottoman cultural life in comparison with the West. Although they are undergirded by Western orientalism emphasizing religion and other cultural features, these Western accounts enrich our understanding of the position of and perceptions around dogs in Ottoman society, since Ottoman texts or narratives do not bring forward or lengthily discuss dogs and other animals that were regarded as ordinarily present in daily life (Obuz, 2022).

**Figure 1:**
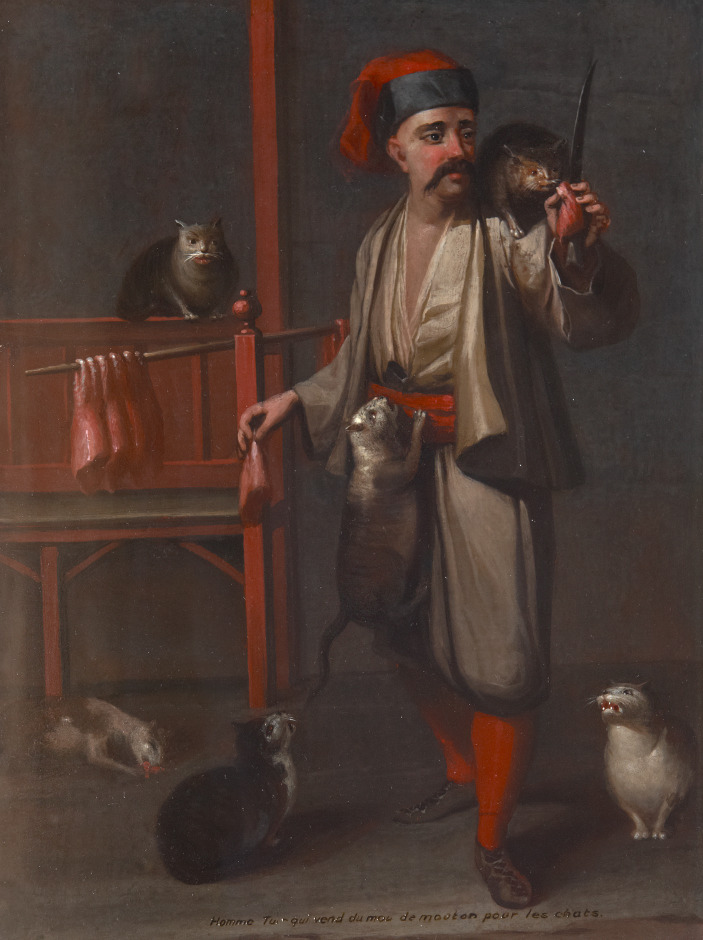
Homme Turc qui vend de mouton pour les chats by Jean-Baptist Vanmour (Valenciennes 1,671¿Constantinople 1737), 1699/1737. A standing man with red and black hat feeding many cats pieces of red meat. Image no. 1010233. Upon special permission of National Trust Collection.

Ogier Ghiselin de Busbecq, Austrian ambassador to the Ottoman Empire in Istanbul in the 16^th^ century, narrates his impressions of the humane treatment of animals among Ottomans. Although regarded as unclean [*necis*] and therefore kept outside of homes (in opposition to cats which were taken inside homes), the Ottomans would still feed the dogs as a charitable act [*sünnet*]^9^ as reported by Forster and Daniell (1881, 225). Another historical account is 16th-century traveler Wratislaw’s narratives of *mancacı,* the profession tending to the feeding needs of street cats and dogs. The word’s origin is traced back to the Italian verb “to eat” [*mangiare*], and the occupation was mostly performed by Albanians (Kuzucu, 2022). A typical *mancacı* would roam the streets of the city with a stick over which he would hang the meat to distribute to street animals (Figure 1). Wratislaw narrates how the city was home to many animals who would sit on the walls, waiting for their alms (Wratislaw, 1996, 65–66). Turkish matrons, Wratislaw narrates, would walk the streets, mumbling prayers, while feeding the animals with tripe and lungs hanging on a stick. Wratislaw himself recounts buying meat from the sellers to feed these animals. Attending to the needs of dogs through feeding in Istanbul was also observed in the cities of Cairo and Alexandria in the 18th century and the beginning of the 19^th^ century (Mikhail, 2015). Back then, it was still common practice to hire people with money to feed and provide for the needs of these animals (Mikhail, 2015). This concern for animals was interpreted as the cultural difference of Ottomans stemming from dogs’ *rightful* claim for compassion, as they are not granted with reason like Man himself, and act on their instincts only (Forster and Daniell, 1881, 225). In Islam, Man occupies a position above all other animals, who are created for him at his service by Allah.^10^ In that sense, Islam continues the human supremacist tradition that is visible in most monotheistic religions (Nocella et al., 2014, xxi). Even religions that are comparably more animal friendly promote kindness to animals from a position that this will eventually result in a higher moral standing for humans, underlining the anthropocentrism in humans’ treatment to animals other than themselves (Nocella et al., 2014, xxi).

However, attentive, and caring treatment toward dogs in historical accounts on Ottoman Istanbul cannot only be explained within rules and observances of monotheistic religions, or Islam in particular. Not only Islam holds a conflicting view on dogs’ status, the history of dogs in the city predates its Islamic governance through Ottoman reign. Recent archaeological excavations of dog remains in Theodosius Harbour of Constantinople (present-day Yenikapı, Istanbul) show evidence of broken dog bones that were healed, pointing at human supervision and care over a certain period of time for these animals that were employed as workers (Onar et al., 2010). These dog bones trace human-dog interaction in the form of care back to Eastern Roman reign in Constantinople predating the Islamic governance of the city in Ottoman Empire. In addition, there remains a vague and ambivalent attitude toward dogs in Islam, thereby causing flexibility and ambiguity in the way interpretations are drawn which reflect the paradigm of the era in question (Mikhail, 2015), also indicating that both generative iterability and indistinctive boundaries are at play. In Islam, the dog represents the soul [*nefs*], which harbours both good and evil deeds, including the potential to be transformed into loyal servants (Gezgin, 2014, 139). Dogs serving human interests are featured in Quran in the story of “Sleepers of Ephesus” or “Companions of the Cave,” who hid in a cave and were protected by the dog Qitmir until the threat of their persecution ended (Robinson, 2018, 82; Gezgin, 2014, 139). Although such companionship offered by a dog to protect devout humans may be expected to elevate the status of the animal in wider Islamic practice, *hadiths*^11^ illustrate rather conflicting views on dogs (Robinson, 2018, 83). Mostly, the dog is considered unclean [*necis*] in different *hadiths* (Pinguet, 2008), that is, the cleanliness of the animal becomes subject to scrutiny. This is in sharp comparison to Quran, which does not problematise the existence of these animals (Gündoğdu, 2023, 30). The most well-known *hadiths* explain that if a dog passes in front of a person praying, the prayer of that person will be cancelled, or that angels will not enter the house where there is a dog (Pinguet, 2008; Robinson, 2018). The main issue with the dog is perceived to be dog’s saliva, which is considered impure and therefore a threat to the ritual purity of the Muslim subject (Abou El Fadl, 2005; Mikhail, 2015). It is believed that the dog’s saliva can contaminate food, carpets, and people’s hands.

**Figure 2:**
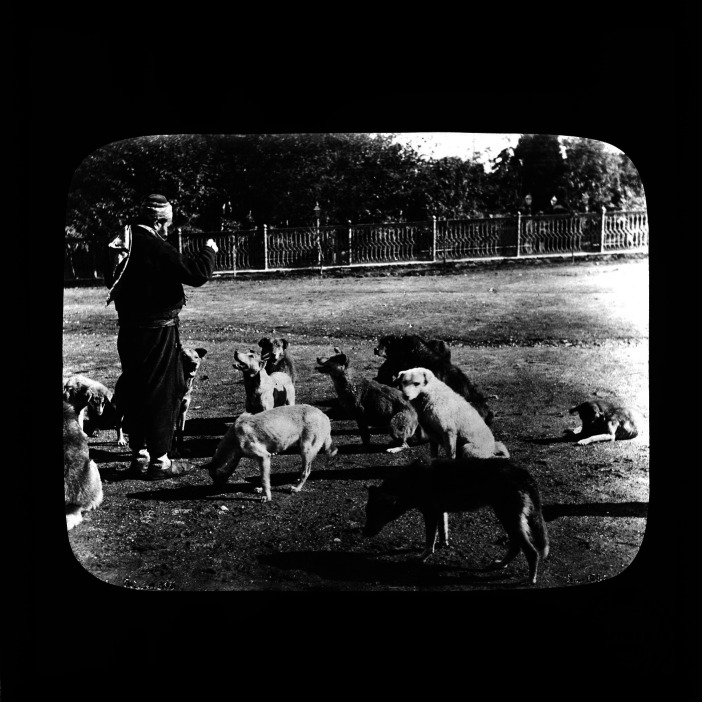
Sokak köpeklerini besleyen bir adam – A man feeding the street dogs. Collection: miscellaneous. SALT Research. https://archives.saltresearch.org/handle/123456789/97238. License: CC-BY-NC-ND 4.0.

**Figure 3:**
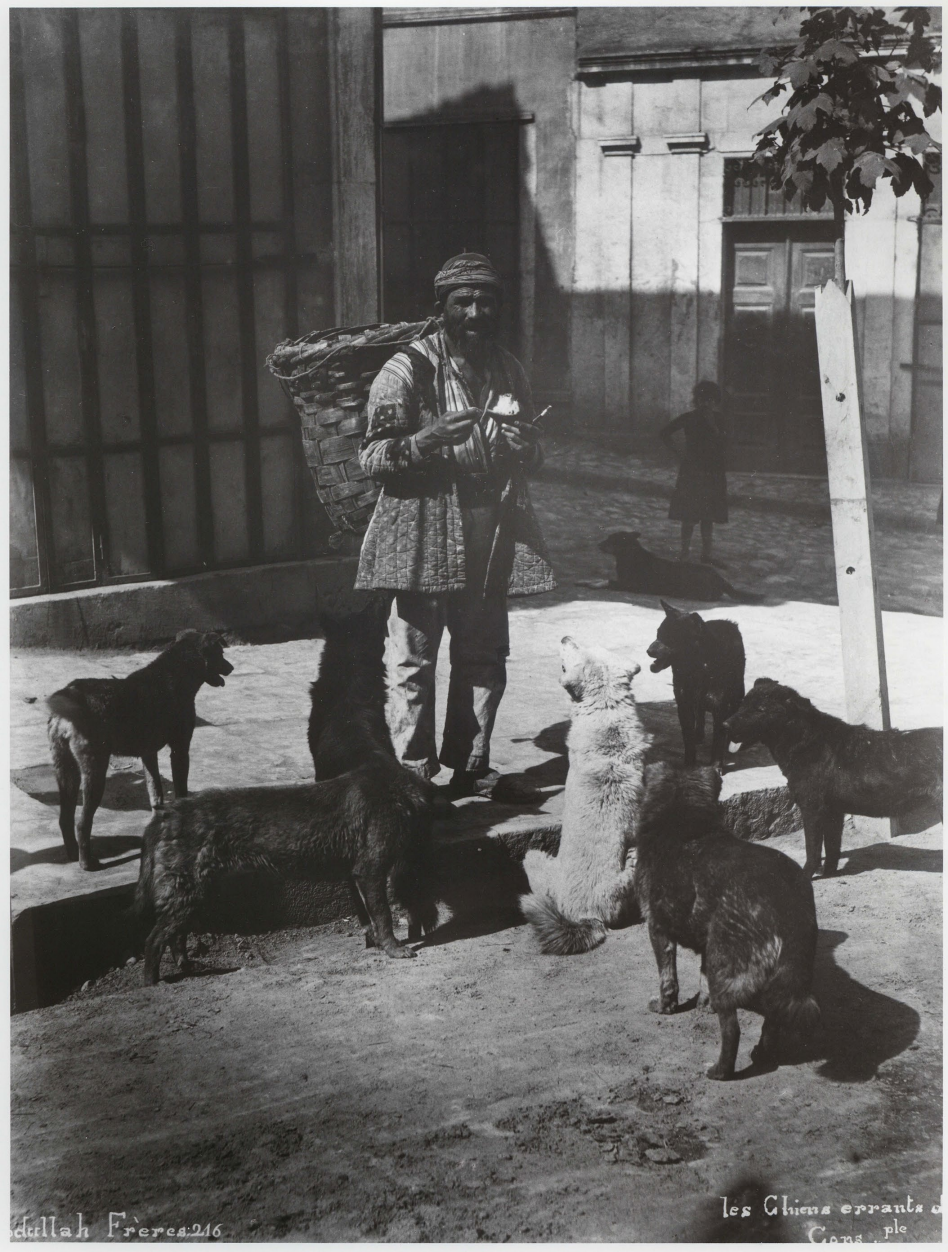
“Les Chiens errants de Constantinople.” İstanbul’da sokak köpekleri ve bir hamal - Street dogs and a porter in İstanbul. Collection: Miscellaneous. Photograph: Abdullah Frères. SALT Research. https://archives.saltresearch.org/handle/123456789/194378. License: CC-BY-NC-ND 4.0.

Beyond the caring narratives stemming from religion to explicate cultural differences in Ottoman Empire, there are also attempts at explaining the functionality of dogs in terms of sustaining urban living during Late Ottoman period. The social and economic position of the dog in urban life is interpreted as a four-legged municipality [*dört ayaklı belediye*] in neighbourhoods mixed with the countryside and partially closed to the outside (Pinguet, 2008). Among the tasks dogs performed were municipal work as both cleaning and sorting the garbage of the neighbourhood, policework as guarding the neighbourhood against outsiders and thieves, and firefighting work as detecting and informing fires, all of which were utilised to help justify dogs’ presence in the functioning of the city (Woods, 1976; Pinguet, 2008; Cox, 2013). Pinguet (2008) argues that dogs’ territorial nature resulted in an organic and reciprocal relationship with the neighbourhood and its residents who provided them sustenance (Figures 1–3). This reciprocal relationship was also preferred by the neighbourhood and wider Ottoman society as evidenced by the sheds built for dogs and other street animals, bequests left for the street animals by deceased wealthy neighbourhood residents, and the foundations [*vakıf*] established for animal protection (Pinguet, 2008).

We argue that, despite being based on observations of dogs scavenging through trash, forming packs, and barking at whoever or whatever they perceive to be a threat, these observations are strategically utilised in an overemphasised manner by those in defence of dogs’ existence in the city. Through an intersubjective lens, however, it could be argued that dogs may simply be nourishing themselves, and reacting to foreign elements, whether that be a passer-by or a fire in the neighbourhood. Justifying the existence of anyone, human or otherwise, through their contribution to productive functioning of the society they are a part of inevitably produces a ranking of deservingness to belong, which we criticised due to its overwhelmingly ableist underpinnings.^12^ We are of the opinion that animals could have functions in interspecies communities (Donaldson and Kymlicka, 2011), and this, in itself, is not necessarily problematic or undesirable. We problematise, however, the lack of a nuanced approach to animal subjectivity through their reduction to mere commodity or labour force. We claim that an excessive focus on the functionality of dogs within Ottoman society in these narratives reduces the subjectivity of dogs to mere social functions and miss the opportunity to further scrutinise the intersubjective relationality that arises from the coexistence of humans and dogs. Later in this manuscript, we will argue that these explanations might be drawing from the Ottoman folk’s exaggeration of such functions to defend dogs during decanisation attempts even though these functions were not as extensive in the past.

## 5 Manufactured distance from street dogs throughout Ottoman modernisation

The systematic problematisation of street dogs in the Ottoman realm commenced as a part of modernisation attempts starting from the 19th century onwards, before the Tanzimat and thereafter (Gündoğdu, 2018; Obuz, 2022). Before the Union and Progress administration and during the reigns of Mahmud II and Abdulaziz, unsuccessful attempts at similar mass deportations of dogs were planned. Late 19th-century traveler notes written by De Amicis (1896, 179) describes these dogs as “poor creatures” who would be mistaken for “mutilated remains of dogs” when they were not moving. A similar impression of dogs could also be observed in Ottoman popular media, in which dogs were regarded as incapable of earning their own bread, who were then likened to late Ottomans being perceived powerless and helpless against the ‘West’, while in others they were linked to the pious Muslim population, who were considered helpless in the face of revolutions (Pinguet, 2008). Such impressions of and feelings toward dogs indicate that what was felt about the dogs was a feeling of pity mixed with symbolic identifications.

Behind these identifications, a mechanism that involves a distancing from these dogs can be revealed. Mary Douglas defines dirt not as germs or filth, but anything that does not fit in the order and system of things (Douglas, 2007, 36–37). Accordingly, ascribing dirt and public menace to street dogs is spatially bound and context dependent. Rather than the dog specie itself posing dirt and/or danger, the association of dirt and danger to dogs indicates a differentiation in terms of dog’s spatial belonging or lack thereof. Dogs of select breeds employed in agriculture in various tasks such as hunting and herding or dogs kept as companion dogs are not the immediate subjects of such bias. It was specifically street dogs who were regarded as dirty, and dangerous, in comparison to dogs who were utilised for their labour and/or companionship. Not only does such employment within and proximity to human interests inevitably rank the animal’s value due to its productivity, but also it indicates *fatalistic normalisation* (Yetiş and Bakırlıoğlu, 2023) within an anthropocentric paradigm encompassing the projections of ableism (dogs without societal functions regarded as naturally dirty, aggressive and dangerous) and racism (favouring certain breeds) onto animals. In this sense, dirt and danger attributed to street dogs, after Samantha Hurn (2012), could be interpreted to function as *distancing devices* that eliminate the empathy required to form intersubjective relations between dogs and humans. While projections of dirt and danger onto street dogs were construed racially and spatially, ‘dog fancy’ phenomenon was spreading in Istanbul, resulting in Ottoman elites joining their European contemporaries in adopting breed dogs as pets (Gündoğdu, 2023), further sharpening the divide between street dogs and pet dogs.

In rapidly growing Istanbul, the need for new spaces and the use of these spaces for public order, infrastructure problems and new economic developments began to change urban structure from top to bottom. In addition, as a result of the centralisation efforts, intervention in the public space by the state accelerated, diminishing the local autonomy of the people, who had used to form partially closed communities within neighbourhoods (Pinguet, 2008). This work is mostly based on the Westernisation-modernisation efforts throughout the Late Ottoman Period and the policies developed as a result of these efforts (Yıldırım, 2021). Undoubtedly, the proclamation of the Second Constitutional Monarchy and the social revolutions that followed in its aftermath presented an image of the Ottoman Empire as the scene of a clash between the old and the new order. However, the topical and ideological nature of these discussions should be noted.

**Figure 4:**
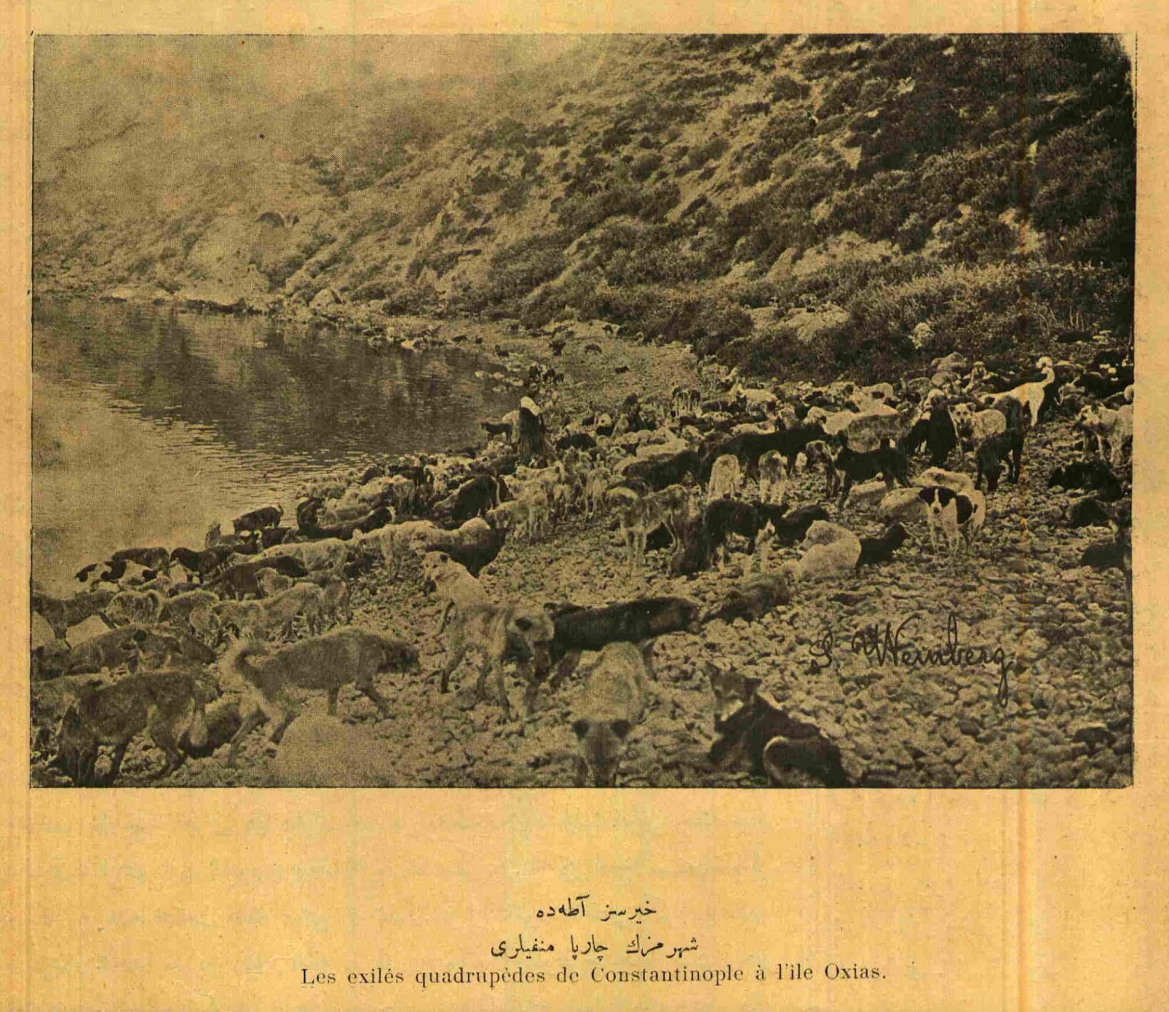
“Hayırsızada’da. Şehrimizin çarpa (dört ayaklı) menfileri (sürgünleri). Les exilés quadrupèdes de Constantinople à l’ile Oxias.” The exile of four-leggeds of Constantinople to the island of Oxias. Photograph: Jean Weinberg. Servet-i Fünun, vol. 995 June 1910, p. 1. National Library of Turkey [*Millî Kütüphane*], Periodicals. https://dijital-kutuphane.mkutup.gov.tr/en/periodicals/catalog/issue/5348.

**Figure 5:**
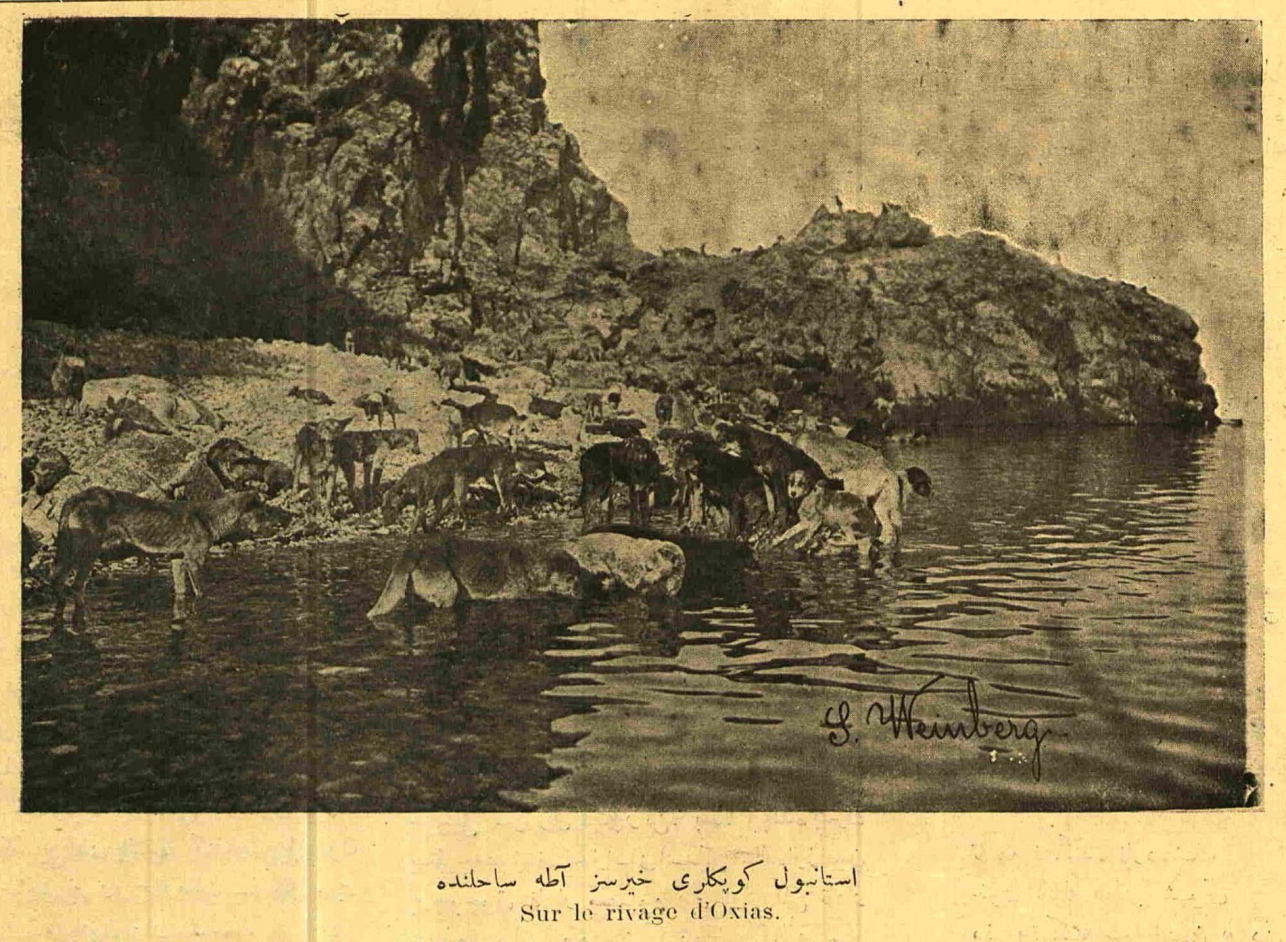
“İstanbul köpekleri Hayırsızada sahilinde. Sur le rivage d’Oxias.” Constantinople dogs on the shore of Oxias. Photograph: Jean Weinberg. Servet-i Fünun, vol. 995 June 1910, p. 9. National Library of Turkey [*Millî Kütüphane*], Periodicals. https://dijital-kutuphane.mkutup.gov.tr/en/periodicals/catalog/issue/5348.

The 1910 Hayırsızada Dog Massacre attracted the attention of both the local and European press. Changes in the public sphere, concerns about the new or criticisms of the old were covered in the press and started generating public debates. Such a systematic massacre made a lot of noise at the time and was met with public reaction. On the other hand, the slaughter of dogs, who were seen by Western travelers as both a nuisance and a symbol of the city, was watched with great interest. Photographs showing the helplessness of the dogs on the island of Oxias [*Hayırsızada*], where they were exiled to death, exemplify this interest (Figures 4, 5). In addition to photography, cartoon, was a popular form of representation, for it allows its author to convey a targeted message, rather than documenting as in the case of photography. Some cartoons appeal to anthropomorphism as a strategy to draw attention to the situation of the dogs. One such example titled “Unlawful Assembly” depicts a pack of dogs, gathered in front of the municipality building, one of whom holds a paper in their mouth, presumably a list of demands from the city governance, in search of justice and compensation on a political ground (Kuzucu, 2022). While the meaning of justice will be discussed more broadly in the following section emphasising its intersubjective dimensions, here we can signify a particular kind of symbolic identification between the traditional folk and street dogs underlined by a shared connection through their claim to the city. In addition, dogs’ unending attempts to return to the city, witnessed by city folk, can be interpreted as dogs’ claim to the city (Kuzucu, 2022). Such symbolic identification can be interpreted as ways of resisting the previously mentioned distancing devices observed in the discourses utilised by the central government as well as the media. However, it can also indicate the resistance against a forgetfulness of the historical co-existence of humans and dogs, and that as age-old residents of the city, dogs’ exile and displacement are simply unacceptable.

Although such symbolic identifications hold the potential to elicit intersubjective relationality, these historical narratives rather reflect a manufactured distance from street dogs onto which dirt and danger were attributed, which we interpret as *distancing devices*. Historical studies on this period mostly interpret such attributions to explicate the displacement and massacre of street dogs as an inevitable outcome of the modernisation process, and the folk’s resistance against these practices of labeling and eradication of street dogs simply as a reaction against modernisation and Westernisation. It is in such historical analyses that dogs are deployed as symbolic resources, which help to explain processes of “human” societies, truncating and then undermining the subjectivities of dogs.

The historical conflict between humans and dogs over urban space manifests itself in the form of various sanctions against dogs in different locales and across different historical periods of the empire (Schick, 2010). In this framework, human treatment of the dog has not changed simply by modernisation and Westernisation, but rather has been historically shaped as a result of a series of contested urban space and resulting spatial reconfigurations. Accordingly, the 1910 Hayırsızada Dog Massacre is not an isolated case, and there were sporadic attempts of killing and deporting dogs throughout the history of the Empire, such as the one in Damascus in the 16^th^ century, the golden age of the Ottoman imperial rule, during which Lütfi Pasha, the governor of Damascus and the brother-in-law of Suleiman I, had about a thousand dogs killed (Schick, 2010). As an example that predates Westernisation processes in the Empire, the Damascus dog massacre illustrates the spatial aspect of these conflicts, rather than the influence of a certain symbolism, be it modernisation or Islamic (Schick, 2010). Two centuries later, in the Ottoman cities of Cairo and Alexandria, dogs were again a significant actor in contestation of urban space. In these cities, the problematisation of street dogs was initiated not by the local population but by French invaders (Mikhail, 2015).^13^

In his article on the 1910 Hayırsızada Dog Massacre, Schick discusses the human-dog relationship through *animal geography* behind the new social order and governmentality that came with modernisation (Schick, 2010). Dogs that were disoriented, hence did not know how to move in this new situation, started to pose a problem for the city people and administration. They were turned into inappropriate creatures of the urban environment, to which they did not quite fit and were construed as dirt (Douglas, 2007). Dogs became dysfunctional with the transition from Istanbul’s more or less autonomous set of self-sufficient neighbourhoods to a metropolis with a high level of urban mobility (Schick, 2010).

After Schick, we interpret human-dog relationship immediately shaped by the changing spatial dynamics of the city, stretching beyond reconfigurations of urban space in modernisation. Dogs lived together with humans in the city as a part of the urban ecology, but with the changing spatial conditions of the city, as well as the European imperial gaze internalised by the Ottoman elites (Brummett, 1995; Gündoğdu, 2018), dogs were labeled as intolerable and discarded by the people they lived with. We conceive human and dog from a relational standpoint and think of these species at different times, in different places, but together. We emphasise the inevitable representation of the animal through a human filter, even without the intention of exploitation or oppression. Our aim is to offer an alternative that recognises the subjectivity of the dog as a fellow creature co-existing with other species, human or otherwise. This perspective paves the way toward a more intersubjective interpretation of such attempts in the historically contested urban space, being wary of undermining animal subjectivity.

## 6 Intersubjective re-interpretation of human-dog interaction in history

Such disorientation in human and animal subjects can be observed in newspaper articles as well. İbrahim Şinasi, a prominent journalist of the period, published an article on 5 May 1864 that spearheaded a modernising discourse that dogs constituted a barrier to a modern city, shared among some members of the urban elite (Şinasi, 1864). The article presented arguments in favour of dogs’ exile and culling. Despite its seemingly anti-dog agenda, Şinasi’s text carried sentimental tones such as pity for the perceived desperation of street dogs, as a result of which he then proposed a ‘solution’.

Two-and-a-half years later, Ethem Pertev Pasha, the then-central governor of Aleppo, published a satire of the social and political problems of the period in Mecmua-i Fünun (Pertev, 1867). The satire is a dialog between a philosopher and a dog named Kıtmir,^14^ invoking Islamic associations to re-articulate the dog in the modernisation process, rather than enacting a conservative stance against modernisation. This imaginary dialog between the human philosopher and the dog is significant for such dialogs constitute the essential basis for anthropomorphism.^15^ In the story, Kıtmir is injured by a carriage and complains about the injustice and ignorance of the humans who want to kill the street dogs as part of modernisation. The philosopher tries to help the dog and offers to write a petition to the authorities on dog’s behalf. The petition is a sarcastic and eloquent plea for dogs’ rights and survival, and a critique of the corruption and hypocrisy of the ruling elite. The dog asks for mercy and justice and proposes a merciful and rational solution to the problem of the dogs’ population and noise. The philosopher suggests that each family should adopt and train a dog, and that the dogs should be spayed during the breeding season. He also joins the defence of dogs based on their role as guardians and cleaners of the streets and questions the cruelty of the logic and morality of the humans who claim to be civilised and superior. Pertev’s emphasis on the functionality of dogs is a continuation of similar exaggeration observed among the folk, as discussed before. However, this indicates that such exaggerated functionality narratives were deployed in order to argue for and defend the co-existence of dogs and humans in modern urban space (Işın, 2021). Such exaggerated accounts on the functionality of dogs demand a change in the form of this co-existence in alignment with the emergent expectations of modern urban living, rather than simply iterating ‘indispensable’ functions of dogs as a part and an extension of human culture and order.

Pertev’s writing is an example of anthropomorphism not in the form of unequivocal anthropocentricism but as a potentiality indicating *generative iterability*. For humans, communicative interaction is a means to influence others, and when such communication is anthropomorphic, it translates to humans constructing the nonhuman entity as an interlocutor in the dialog. This imaginary dialog, then, translates to establishing a relation with the nonhuman entity (Airenti, 2018). In his article, Pertev iterates human capabilities, and concepts toward the dog, such as writing, communication, justice, and politics. However, this iteration does not merely copy human capabilities and concepts onto dogs, but calls for a transformation with a difference, as the dog’s perspective and situation are translated into a human narrative which does not only reflect human concerns but also animals’ concerns as symbolic actors rather than resources. In such cases, it is of significance that we regard anthropomorphism as a potentiality with a creative and adaptive response to the challenges of existence and crisis-driven situations (Jackson, 1998) rather than dismissing it due to its anthropocentric origins. Anthropomorphism, then, goes beyond human’s attribution of and identification with dogs and becomes a catalysing instrument to help establish an intersubjective ground between dogs and humans other than the author themselves. Anthropomorphism is appealed to evoke a certain affect and a call for justice, by which the author attempts each individual reader regards the dogs as subject.

Various forms of resistance to dogs’ mass exile in the city were also observed among the folk. In the 1830s, the order of Mahmud II to deport the dogs to the island following an incident where a British citizen died while being chased by the dogs in Beyoğlu, Istanbul (Kuzucu, 2022), resulted in such a resistance among the Ottomans that the Sultan had to withdraw its order. Such resistances also involved peculiar interpretations of social events, which require elaboration. For instance, some of these planned exiles could not be realised due to natural disasters, and this was interpreted by the public as the wrath of Allah for the treatment of dogs (Pinguet, 2008) as a sign of poetic justice. The folk also attributed the loss of the second Russo-Turkish War (1828–1829) to the mistreatment of dogs in Istanbul (Kuzucu, 2022). Such historical events pertaining to the deportation of dogs cannot be reduced to folk’s resistance to modernisation processes *per se* or attributed to remnant cultural features of traditional Ottoman culture. They rather indicate the *iterability* of these features for Ottoman folk to convey their feelings of discontent and lament and even to demonstrate resistance in consideration for fellow dogs. Still, even if these accounts partially reflect the intersubjective relationality of humans and dogs through the *generative iterability* of religious and cultural scripts and anthropomorphic narratives, they remain limited in reflecting the dog’s agency in the constitution of this relationality.

Beyond iterability, the *arrivant* nature of this intersubjective relationality of humans and dogs should be unfolded to reveal the agency of the dog. A personal story of Samipaşazade Sezai, a renowned writer and diplomat from Istanbul in the Late Ottoman period, writes on his relationship with a street dog who follows him everywhere (Sezai, 1898). Sezai initially finds the dog both annoying and amusing, as the dog (even when the dog is wounded) waits for him when he enters buildings, or when the dog barks at strangers approaching him. One day, when contemplating on his relationship with a friend who is upset with him, Sezai suddenly notices the presence of the dog right next to him. He comes to the realisation that the dog is a true friend who protects him and waits for him patiently. He feels ashamed of his neglect of and indifference toward the dog and decides to apologise to him. In this account, the insistent attitude of the dog who consistently follows Sezai stands for an *arrivant* nature that both interrupt the familiarity of an established positionality between a street dog and a fellow human embedded in the cultural/historical script and is resistant to be interpreted in the same way as if it happens between two human subjects. If he were to be followed by a human subject everywhere, Sezai’s interpretation would probably be different. Hence, in this specific story, it can hardly be claimed that either another dog can replace/substitute the human subject and vice versa, or another human subject can invoke the very same affection as the dog does through such interaction. Dog’s unconditional loyalty as interpreted by Sezai, as well as the unanticipated care in-between the human and the dog indicate a reciprocal relation of mutual recognition and respect imminent to joint attendance, which is initially dismissed by the author. This story presents a doubly binding intersubjective relationality. While the boundary remains here as a mediating space between the subjects, it also harbours an indistinctive character since it can be blurred, transgressed, and re-interpreted continuously through the dynamics of intersubjective relationality.

Another historical account sheds a different light on such intersubjective relationality, in the sense that it emphasises dog’s agency in shaping human-dog interaction in the face of emergent conditions, going beyond dog as a symbolic actor. The origins of the dog colony that is believed to exist in Rumelihisarı, Istanbul to this day is such an example. In a newspaper article titled “The Constantinople Dogs” (1910), the vice-principal of Robert College in Istanbul, Dr. Albert Long, adopts a large, beautiful, and intelligent dog to guard his house and garden, named Karabaş. Karabaş is protective against beggars and strangers but greets well-dressed guests. One day, Dr. Long notices that Karabaş is lying on the snow instead of his shed, in which there is now a small, female street dog. He chases away the street dog for several days, and each time, in an increasingly harsher manner. Finally, Karabaş leaves with her and settles near Rumelihisarı. The professor and his family are initially surprised by this, but upon realising that Karabaş would not return home, they still care for the dogs and their puppies, and Karabaş ends up building a new life for himself. This example, again, reflects the *arrivant* nature of the intersubjectivity regarding its unanticipated emergence and the indistinctive character of boundary that is similarly interrupted, blurred, and reshaped through the dynamics of such relationality.

Both Sezai’s account with the street dog who follows him everywhere, and Karabaş’s establishing a dog colony with a female street dog in Rumelihisarı illustrate the emergent and dynamic character of intersubjective relationalities of humans and dogs shaped through *indistinctive boundaries*. In the former, a human develops familiarity and intimacy through joint attendance with a street dog that he initially describes as indifferent and ordinary. In the latter, the street dog, a liminal being, pursues a rather feral way of living, as a result of which the human-dog interaction takes a different shape, where human subjects are drawn into the formation of a dog colony. These historical accounts demonstrate how the *indistinctive boundaries* approach we articulate can reveal the diversity and complexity of human-animal relations in different contexts.

## 7 Conclusion

In pursuit of ways to uncover the continuity of intersubjective relationality and co-existence of humans and animals throughout history, this article particularly focuses on the case of human-dog relations in Istanbul during the Late Ottoman period in recognition of the repercussions of modernisation processes, especially for street dogs, whose existence were repeatedly problematised and who were exiled en masse in 1910. The period also witnessed discomfort and even resistance to such implementations. We argued that this should neither be reduced to remnants of “traditional” Ottoman culture interpreted as hospitable toward and accommodating of animals, nor should be seen as a mere extension of the Ottomans’ resistance against modernisation attempts. Rather, we argued that these reflect both continuous and changing dynamics of intersubjective relationality between street dogs and people, complicating the story which we tried to relay.

Using various sources (i.e., Western travelers’ travelogues, Ottoman popular media, articles and diaries) that were thematically (i.e., human-dog interactions) selected that illustrate how street dogs were perceived and presented by historians and other social scientists, we attempted to illustrate how the peculiar forms of intersubjectivity between humans and dogs has been truncated if not totally denied, forgotten or excluded in historical narratives. However, despite such truncation and denial, intersubjective interactions between humans and dogs in historical sources can be noticed upon closer scrutiny. In uncovering human-dog intersubjectivity, we utilised a theoretical framework consisting of four pillars: (1) Jacques Derrida’s *différance*, *iterability* and *arrivant,* (2) Matthew Calarco’s *indistinction* in human and nonhuman animal coexistence, (3) Jessica Benjamin’s *mediation* based on her conception of intersubjectivity, and (4) Donna Haraway’s *companion species* that highlights the interdependency and co-evolution of human and dogs. In understanding and discussing the human-animal divide, we merged Derrida’s *différance* and Calarco’s *indistinction* approaches and offered “indistinctive boundaries,” an approach that is attentive to the species differences so that it constitutes “inter,” but also flexible and indeterminate that this divide may be rendered indistinctive. Donna Haraway’s notion of companion species helped combat traditional notions of dog domestication and human-dog co-existence through the lens of interdependence and co-evolution, mediated through time and space, after Benjamin, through which one may be able to read the intersubjectivity of both. Although the original concept is not intended for human-animal interaction, we propose that Benjamin’s notion of intersubjectivity holds potential to understand human-dog relations, for interdependence is a constituent aspect of such relations. Therefore, we aimed to demonstrate how a close reading of historical accounts through the lens of human-dog intersubjectivity reveals the agency of dogs in history and go beyond anthropocentric interpretations as best as possible. We are aware that this is quite a challenging and impossible task, yet intersubjectivity as an analytical concept offers an alternative path.

We strived to reinterpret the historical accounts to trace and unearth the continuity of intersubjective relationality and co-existence of humans and dogs in the Late Ottoman period. In doing so, we argued that the functionality attributed to dogs in sustaining the city (e.g., municipal work, policework, firefighting work) were actually animal subjects’ forms of interaction with fellow humans as well as external stressors in their neighbourhoods as continuing processes of co-evolution. Such functional roles, we attested, were strategically exaggerated by Ottomans to justify the existence of dogs in the city in the face of decanisation attempts. In a similar vein, anthropomorphism was *generatively iterated* by period writers and thinkers as well as by ordinary folks to translate dogs’ experience into a human narrative. The *arrivant* nature of dogs has disrupted the founded familiar positionality between a street dog and a fellow human and posed alternative forms of intersubjective relationality. All these point to the continuity of co-existence and intersubjective relationality throughout history, which was implicitly or explicitly disregarded in most historical narratives.

It should be noted here that the co-existence we reveal is limited to Late Ottoman Istanbul. This human-street dog co-existence is still strongly visible in the city, a distinctive quality of the urban environment, which is very different from the urban contexts of the Global North. Deploying intersubjectivity in re-interpreting the histories of other locales would yield different results. In addition, our analysis is limited to human-dog relationality, and cannot be directly translated to other intersubjective relationality among humans and other animals, domestic or otherwise. Studying interspecies intersubjectivity requires attending to the specificities of spatial and temporal contexts. It should be noted that intersubjectivity, while valuable, gains meaning within spatial and temporal contexts through *mediation*. Hence, we argue that its utilisation should be contextualised, and it should not strive to draw generalisable conclusions.

In order to develop a nuanced understanding of animal subjectivity, which we may never fully grasp (Horsthemke, 2018) but may only strive to attain, we propose human-animal intersubjectivity as an analytical concept. Due to the unavailability of direct, empirical observation of animal subjects in historical contexts, we argue that intersubjectivity is especially fruitful as an analytical concept to interpret human-animal interaction in historical accounts. When intersubjectivity between human and nonhuman animal is disregarded, it is inevitable that animal’s subject status is diminished, resulting in a truncated picture of the animal reality.

Even though we underline the importance of human and nonhuman animal co-existence, it is nonetheless vulnerable and not guaranteed. That’s why we argue for the importance of recalling the continuity of intersubjective relationality among human and nonhuman animals and co-existence by emphasising the aspects of joint attendance including mutual recognition and respect which are prominently important for political and ethical endeavours to be taken – even if it is continuously changing in mostly unpredictable ways. As such, we are proposing an alternative approach to trace the continuity in historical text, which we believe can be beneficial for other scholars, activists, and practitioners in pursuit of similar traces of human-animal interaction throughout history.

## Data availability statement

The original contributions presented in the study are included in the article/supplementary material, further inquiries can be directed to the corresponding author.

## Author contributions

BT: Writing – original draft, Writing – review & editing. EY: Writing – original draft, Writing – review & editing. YB: Writing – original draft, Writing – review & editing.

## Footnotes

^1^This fictive narrative is not accurate from a historical perspective, as the exile of dogs happened in 1910 whereas Sultan Abdulhamid was overthrown the year before.

^2^Here it should be noted that we do not necessarily liken domestication to slavery, as we will discuss later, but draw parallels between the two phenomena in terms of the interdependencies that could be observed in both.

^3^From here onwards, we refer to nonhuman animals simply as “animals” for the sake of legibility.

^4^We remain cautious of accounts that ground animal subjectivity based on the cognitive capacities of animals or lack thereof, one example to which, we believe, is joint attention. As a result, we use ‘joint attendance’ as a more encompassing approach to describe how individuals in a social interaction, human or nonhuman, attend to each other’s existence, and respond accordingly. Such an approach rejects ableist-speciesist nexus, as aptly described Muller and McNeill (2021), and looks to the linked oppressions among animal and disabled bodies (Taylor, 2017). Such an approach chooses to reject humans as well as nonhuman animals based on their proximity to abled human, inevitably resulting in a peculiar ableist anthropocentrism. Joint attendance delineates firm distance to human-centric cognitive and/or emotional definitions, although remaining aware of the necessity of incorporating emotions as a feminist agenda in dealing with nonhuman animals. Such attendance to other ways of being has been elaborated by scholars across humanities (Tsing, 2015; Desai and Smith, 2018). We argue that joint attendance is the basis for interspecies co-existence as a politico-ethical requirement to ensure animal justice.

^5^Symbolic interactionist approaches emerging in the 1990s to include the animal as a symbolic actor (e.g., Arluke and Sanders, 1996; Alger and Alger, 1997; Irvine, 2012) are preceded by the systematic and dedicated study of the nonhuman in Science and Technology Studies (STS) in the 1980s, especially visible in Actor-Network Theory (Callon, 1984; Latour, 1987). While Actor-Network Theory explicitly grants agency to the nonhuman (animal or otherwise) beyond as symbolic actors, it is not concerned with subjectivation processes, but with how actants (human and nonhuman) within networks shape social processes and outcomes. Since our focus in this paper is intersubjective relationality in human-animal interactions, we do not elaborate Actor-Network Theory or similar approaches.

^6^As the reader might notice, this approach approximates to the identity-based approach criticised in the previous section.

^7^After Losey (2022, 133), we use the word “domestic,” instead of “domesticated” to underline that domestication is an ongoing process (Zeder, 2012a; Losey, 2022).

^8^Yıldırım (2021) refers to decanisation as a unique phenomenon in the sense that it disrupts extant human-dog interaction and mutual co-existence compared to dog culls across history the aims of which were to maintain control over the dog population in the city and the spread of zoonotic diseases.

^9^Sünnet, or sunnah, refers to the traditions and practices of the Prophet Muhammad, which are considered a model for Muslims to follow.

^10^Quran 36:71–72.

^11^Hadiths are the recorded sayings and actions of the Prophet Muhammad and is one of the main sources of Islamic law and theology, along with the Quran. Sunnahs are derived from hadiths.

^12^This is a point that is discussed also within critical disability studies. Due to the scope and limitations of this paper, we do not go into details.

^13^These dogs, who were constantly on the offensive against these newcomers, orchestrated fierce resistance against Napoleon’s armies to enter the inner parts of the cities and their narrow streets, putting the French army in a difficult situation (Mikhail, 2015). Not expecting such opposition, the French army took to the streets at night and poisoned the dogs, and the local population faced a mass slaughter of dogs during the daytime (Mikhail, 2015). The French invasion brought its own governmentality strategies to Egypt and many regulatory practices, accompanied by the development of public measures against dogs, including exile, slaughter, and deserting in islands (Mikhail, 2015).

^14^Kıtmir is an iteration of the name Qitmir, the dog mentioned in the Quran.

^15^One of the ways in which anthropomorphism occurs is attribution of human-like cognitive capacities to animals. There are three main factors that drive such anthropomorphism, which are the perceived similarity of the animal to the human, the group membership of said animal in terms of evolutionary trajectory, and if applicable, the interspecies bond between subjects (Eddy and Povinelli, 2010).
